# Muscle-Derived Cells for Treatment of Iatrogenic Sphincter Damage and Urinary Incontinence in Men

**DOI:** 10.1100/2012/898535

**Published:** 2012-07-31

**Authors:** H. Gerullis, C. Eimer, E. Georgas, M. Homburger, A. G. El-Baz, M. Wishahi, M. Borós, T. H. Ecke, T. Otto

**Affiliations:** ^1^West German Cancer Center (WTZ), University of Essen, Essen, Germany; ^2^Department of Urology, Lukas Hospital Neuss, Preussenstraße 84, 41464 Neuss, Germany; ^3^German Centre for Assessment and Evaluation of Innovative Techniques in Medicine (DZITM), Neuss, Germany; ^4^Department of Urology, Theodor Bilharz Research Institute, Cairo, Egypt; ^5^Department of Experimental Surgery, University of Szeged, Szeged, Hungary; ^6^Department of Urology, HELIOS Hospital, Bad Saarow, Germany

## Abstract

*Introduction*. Aim of this study was to assess the safety and efficacy of injection of autologous muscle-derived cells into the urinary sphincter for treatment of postprostatectomy urinary incontinence in men and to characterize the injected cells prior to transplantation. *Methods*. 222 male patients with stress urinary incontinence and sphincter damage after uroloical procedures were treated with transurethral injection of autologous muscle-derived cells. The transplanted cells were investigated after cultivation and prior to application by immunocytochemistry using different markers of myogenic differentiation. Feasibility and functionality assessment was achieved with a follow-up of at least 12 months. *Results*. Follow-up was at least 12 months. Of the 222 treated patients, 120 responded to therapy of whom 26 patients (12%) were continent, and 94 patients (42%) showed improvement. In 102 (46%) patients, the therapy was ineffective. Clinical improvement was observed on average 4.7 months after transplantation and continued in all improved patients. The cells injected into the sphincter were at least *~*50% of myogenic origin and representative for early stages of muscle cell differentiation. *Conclusions*. Transurethral injection of muscle-derived cells into the damaged urethral sphincter of male patients is a safe procedure. Transplanted cells represent different phases of myogenic differentiation.

## 1. Introduction

Approximately two hundred million people worldwide are affected by urinary incontinence [1]. The condition may significantly reduce quality of life and is known to exacerbate comorbidities [2]. Stress urinary incontinence (SUI), characterized by an involuntary passing of urine, related with exertion, sneezing, or coughing is considered the most common form of the disease [3]. 

Etiology for SUI may be different in women and men. In female patients, functional impairment of pelvic muscles, connective tissue and their associated innervating nerves, conditions likely to occur secondary to pelvic floor damage following vaginal childbirth may trigger SUI as well as advancing age, obesity, and hormonal status [4]. It is currently assumed that the majority of patients presenting with SUI presents combined elements of intrinsic sphincter deficiency and hypermobility [5]. SUI in men due to damage of the external urethral sphincter during surgical procedures, mostly radical prostatectomy, is a rare but well-known complication. Depending on the severity of postoperative stress urinary incontinence and on the patient's desire, various treatment options can be offered, covering non-invasive therapies, pharmacological treatment approaches, surgical treatment including injection therapy (bulking agents), application of sling-systems and cell therapy [6]. All of these methods have limitations and new, innovative experimental approaches are being proposed to improve treatment success. Currently available reports of clinical experiences with implantation of muscle-derived cells into the subvesical sphincter have been motivated by preclinical studies proving feasibility of the method and showing myoblasts to have potential for regeneration of the rhabdosphincter [7–9]. 

Injection of muscle-derived cells into the rhabdosphincter in patients with SUI has been repeatedly reported; however, clinical studies and observations predominantly refer to female patients [10, 11]. Some of them are controversially discussed [12]. 

In the only available study referring to male patients, Mitterberger and co-workers injected autologous fibroblasts and myoblasts, under ultrasound guidance, into the sphincter of 65 patients with postprostatectomy SUI and reported a therapy respondence rate of 89% after 1-year follow-up [13]. 

In the present study, we describe our results regarding feasibility and efficacy of injecting muscle-derived cells (MDC) into the urethral sphincter of 222 men with refractory postoperative SUI due to sphincteric damage after urosurgical procedures.

## 2. Material and Methods

### 2.1. Legal Background

Based on a special permit of the regional government of NRW, we offered our patients MDC cell therapy as an “individual treatment attempt.” This permission was temporarily limited from 2005–2011. On this base, with the exception of serological tests for HIV and HBV exclusion, patients were not required to pay for any of the performed diagnostic and therapeutic procedures as the costs were covered by our institution. Preparation and transplantation of cells corresponded to §4a AMG (Germany). For the entire procedure of diagnostic, cell harvesting, tissue engineering, and transplantation, we developed 32 standard operating procedures (SOPs) according to current GMP/GCP guidelines. Helping them to reach a decision on their own, all patients were extensively enlightened about the experimental character of the therapeutic approach. All patients had to sign an informed consent before receiving the therapy.

### 2.2. Patients

From 2005 to 2011, 222 men (mean age: 70 years; range: 56–81) were treated and studied. Patients originated from entire Germany.  Table 1 displays the most relevant patient characteristics. As inclusion criteria for treatment we defined: (a) SUI grade III (auxiliary means: diaper trouser and condom urinal), (b) residual-free bladder emptying, (c) duration of incontinence >12 months, (d) therapy-refractory urinary incontinence (physiotherapy, pelvic floor work out, electric stimulation, and duloxetine), and finally (e) endoscopic proof of a sphincteric lesion. As exclusion criteria, we defined (a) previous irradiation and (b) previous application of bulking agents. The iatrogenic sphincter defect was the consequence of radical prostatectomy (*n* = 192), transurethral prostate resection (*n* = 9), or radical cystoprostatectomy with neobladder (*n* = 21). All patients had a lesion of the external sphincter, which was documented under endoscopy. The distribution of the sphincter damage in these patients having undergone radical prostatectomy has been recently reported and seemed to follow a particular local distribution pattern [14]. 

### 2.3. Tissue Engineering

Under general anaesthesia, endoscopic evaluation of the urethra and the urethral sphincter were performed. When indication for MDC treatment was given, we took within the same surgical procedure, four skeletal muscle biopsy specimens (5 × 5 mm each) from the deltoid muscle of the contralateral side of the preferred hand through a 15 mm skin incision. The harvested material was immediately brought into 10 mL sterile transport solution (Liforlab, Lifeblood Medical) and subsequently transported to our laboratory for tissue engineering and kept at room temperature for 24 h. Then, the biopsied muscle was minced into a coarse slurry using razor blades. After mechanical shearing using a scalpel, the homogenate was centrifuged, the cells were plated in a proliferation medium consisting of DMEM/F12, 10% fetal calf serum, 5% glutamine, and 5% penicillin/streptomycin. Cell culture was maintained until a quantity of at least 10 × 10^7^ cells.

### 2.4. Immunocytochemistry

In order to learn about the characteristics of the transplanted cells, we performed immunocytochemistry after successful culturing priorily to transplantation. Therefore, cells grown on chamber slides were rinsed in PBS, fixed in 3.75% paraformaldehyde/PBS for 15 min at room temperature. Fixed cells were incubated in 0.1 M NH4Cl for 10 min, followed by incubation in blocking buffer consisting of 1% BSA and 0.1% Triton X-100/PBS for 15 minutes. Slides were incubated for 30 min with primary antibodies diluted in blocking buffer. The primary mouse antibodies were *α*-sarcomeric actin, *α*-smooth muscle actin, desmin (all from DakoCytomation), MyoD1 (Sigma-Aldrich, Inc.), and CD34 (Dianova). Cells were washed three times for 10 min in PBS and were then incubated for 30 min with a fluorescein- or CY3-conjugated secondary antibody (Jackson ImmunoResearch) in the dark. Nuclei were counterstained with 49.6 diamidino-2-phenylindole (DAPI). After mounting in Fluoromount-G (Southern Biotech), cells were imaged on an Olympus BX51 microscope equipped with a AxioCam HRc camera (Zeiss), and images were acquired using AxioVision 4.4 (Zeiss). Negative control stainings were performed using nonspecific mouse IgG (Jackson ImmunoResearch) and IgM (Dakocytomation), respectively.

### 2.5. Autologous Cell Transfer

Transplantation of cultivated MDC was planned after successful cell culture and expansion of cells and finally performed after achievement of a confluent growth in culture under endoscopic view circumferentially to the specific defect of the sphincter. A minimum of 5 injections around the sphincter damage was performed.

### 2.6. Assessment of Treatment Success

This was not a clinical study but a treatment attempt as previously declared to each patient. Patients were not requested to participate in any additional examination. Therefore, and due to the fact that patients originated from the entire country, we tried to simplify the re-evaluation process as much as possible. Thus, evaluation of treatment success was done by simply asking the patient or the respective treating urologist who was involved in follow-up. For simplifying purposes, we defined two types of questions: (1) incontinence status: (a) still incontinent, (b) improved, or (c) continent and (2) if effective: when was improvement seen first? Side effects possibly related to the procedure were assessed accordingly? We considered those questions as sufficient for primary evaluation of treatment success and easy enough to be performed with regard to the limitations of the predescribed setting. We asked the patients or respective urologists earliest 6 months after autologous cell transfer.

## 3. Results

### 3.1. Functional Results

We received information regarding treatment success from all included patients or the respective office based urologists. The average time since iatrogenic sphincter injury was 43 months (range: 12–192). All patients were followed-up for at least 12 months after MDC transplantation using the predescribed methodology. 26 patients (12%) were continent, 94 patients (42%) stated improvement from their initial urinary incontinence. 102 patients (46%) reported persistent urinary stress incontinence without any improvement after therapy. Clinical improvement was observed on average after 4.7 months (range: 2–9) and continued in all improved patients (observation period: 12–54 months). Complications of the initial surgery were protracted catheter insertion for 3 days due to hematuria (*n* = 4), peri-interventional cystitis (*n* = 11), and further impairment of the preexisting urinary incontinence by removal of the stricture (*n* = 19). 

Reversible, minor side effects were observed in 12% (26/222) of patients: perineal pain (*n* = 11), orchidoepididymitis (*n* = 6), urethritis (*n* = 5), and mild fatigue syndrome (*n* = 4). A positive effect after transplantation of cells into the damaged sphincter was seen not earlier than after 2 months. On average, 5.2 × 10^6^ cells were injected to patients (range: 1.2–19.2 × 10^6^). The mean duration of primary cell culture from harvesting to transplantation was 61 days (16–122).

### 3.2. Cell Characterization

**Figure 1:**
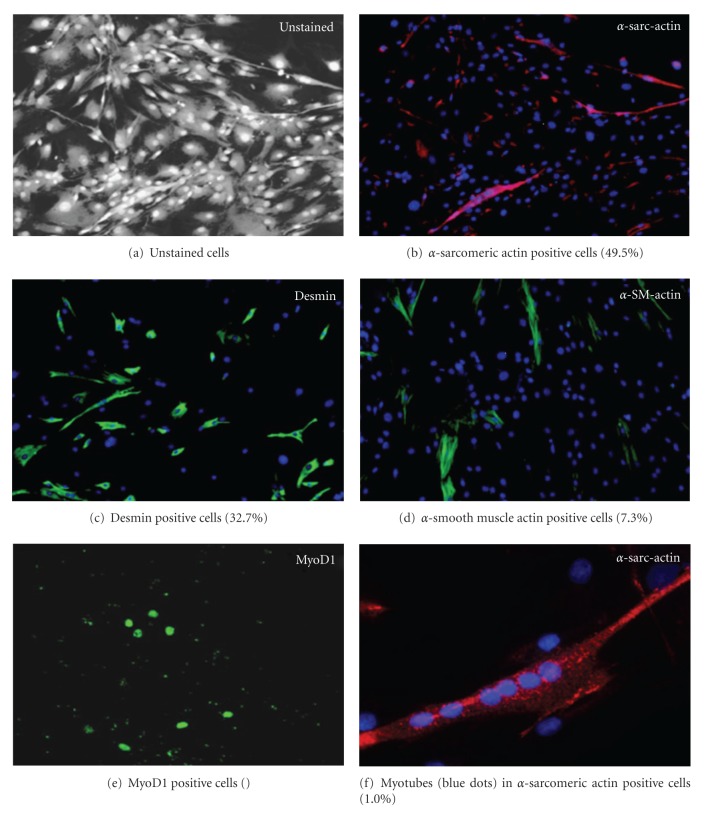
Immunostaining of cells prior to transplantation.

To investigate the nature of the transplanted cells, skeletal muscle-derived cells were investigated by immunocytochemistry for the expression of different markers involved in muscle cell development and differentiation (Figures 1(a)–1(f)). Light microscopy revealed populations of cells different in size and shape. From these, 49.5% (SEM 2.8) were positive for *α*-sarcomeric actin, 7.3% (SEM1.8) for *α*-smooth muscle actin, and 32.7% (SEM10.1) for desmin, respectively. Co-immunostaining experiments demonstrated that 2.8% (SEM 0.5) of the cells were positive for both *α*-sarcomeric actin and *α*-smooth muscle actin. The myogenic transcription factor MyoD1 was present in some but not all patients (0%–9.1%), while no CD34 positive cells could be detected. 

## 4. Comment

Stress urinary incontinence after surgical procedures, mainly radical prostatectomy, represents a functional complication with highly significant impact on patients' quality of life. It can be caused by lesions to the external urethral sphincter [14]. In this study, we report on 222 incontinent male patients with damage to the external sphincter which have been treated by transurethral injection of autologous muscle-derived cells. To date, only one study is reported for using MDC selectively in men [13]. Mitterberger et al. used a transurethral ultrasound guidance for application of myoblasts and fibroblasts. This was necessary as they intented to distribute the cells equally to all parts of the sphincter. In their study, post-RP incontinence has been interpreted as being caused by loss of rhabdosphincter cells and consecutive reduced function of this muscle, finally resulting in SUI. In contrast, we have assumed a particular damage to the sphincter as being responsible for the post-surgery incontinence; thus in contrast to the Austrian researchers, the application of cells was directed to the circumference of this endoscopically identifiable area and did not mandatorily require additional sophisticated devices. This is in accordance with other reports which injected cells under endoscopic vision only [10]. Indeed, we agree that successful endoscopic injection of cells is highly dependent on the surgeon's skills and should therefore be performed by experienced urologists with high volumes of respective endoscopic procedures. In comparison to Mitterberger et al. who were able to include the high number of 63 post-RP incontinent patients in one single year, we needed almost 6 years to reach the number of 222 post-RP incontinent patients, also recruiting from different surgical centres of one country. This difference may result from the different inclusion criteria, as an endoscopically proven sphincter damage was mandatory for inclusion into our cohort.

The majority of available studies with muscle-derived or stem cells report on application in women where the etiology of SUI is different from men with defined sphincter damage after surgery. In our opinion it seems very unlikely that cell therapy is able to have a comparable effect as lifting up entire organs/tissue with slings or more invasive surgical procedures which aim to reinforce the weakness of pelvic floor muscles and supportive ligaments or fascia. However, the reported results are highly optimistic. Muscle cell or stem cell injection into the middle urethra seems to have potential to restore the contractile response of the striated muscle and rhabdosphincter. 

To date, cell therapy seems not to be the new ultima ratio for long-term incontinent patients, but rather an interesting approach for a smaller patient group, which is not yet identified as pathology of SUI is not entirely understood and varies among patients. Male patients after surgery with normal pelvic floor function and a defined sphincter damage may be possible candidates. This thesis is strongly underscored by a recent report from Eberli and co-workers who employed a model of irreversible sphincter damage in dogs, resembling the clinical situation of an accidental surgical damage [15]. The authors could show formation of new muscle tissue and improvement of sphincter functionality for up to 6 months. Intact innervation and blood supply in sphincters with a defined damage were interpreted as important conditions for the success of this therapeutic approach. The authors concluded that injection of autologous MPCs into irreversibly damaged sphincter muscle could be an appropriate indication. We agree with Eberli et al., although our study has been observational in nature; both approaches rely on the same indication and would warrant further investigations as suggested.

It remains critical to evaluate what a predefined amount of injected cells is able to perform in a destroyed or weak sphincter. The processes are not understood; however, we can imagine that injection of MDC into a predescribed anatomic and functional defect may be of functional benefit. Therefore, sophisticated imaging procedures and methods in vivo and ex vivo have to be evaluated in order to trace the injected cells. The animal model suggested by Eberli et al. may be a very helpful tool for first investigations regarding the data reported here [16]. 

In our patient group, a clinical effect was seen earliest after 4–6 months following cell injection, leading to the assumption of a sustainable functional effect on muscle regeneration of the applied therapy. As no effect was seen within the first three months, a bulking effect was considered very unlikely. In contrast, the described temporal delay is more consistent with repair of the muscular defect, and subsequent re-innervation also underscored by the characteristics of transplanted cells as shown in this study. 

A number of prior studies have reported repair of urinary incontinence in experimental animals and give possible explanations for the observed treatment success. A significantly increased subvesical resistance is reported in dogs after application of autologous muscle cells into the external sphincter (after prior microsurgical diminution by 50%) [17]. Zeng reported the restoration of urethral function after microsurgical transabdominal urethrolysis by pluripotent human fat cells which interface with carrier molecules in immunodeficient rats [18]. Urinary continence by skeletal myoblasts which also provided guiding structures for re-innervation in female pigs was re-established by Lecoeur and colleagues [19]. 

In the current study, the identity of the transplanted cells was partially characterized by immunostaining for different markers involved in muscle cell development and differentiation. Approximately, 50% of the cells of all patients were positive for the skeletal muscle marker: *α*-sarcomeric actin. Only ~7% were positive for *α*-smooth muscle actin, a marker for differentiating smooth muscle cells or myofibroblasts [20, 21]. The few cells double positive for *α*-sarcomeric actin and *α*-smooth muscle actin were presumably cells caught in the course of skeletal myoblast differentiation [22, 23]. Desmin is another marker for skeletal and smooth muscle cells, abundantly present in our patients (~30%), which satellite cells are known to start expressing at the beginning of myogenesis [24]. The transcription factor MyoD is another early marker of myogenic differentiation and proliferation expressed in satellite cells early in skeletal muscle development [25]. Very few or none (0%–9%) of the cells expressed MyoD, suggesting that there are none or few satellite cells in this stage (either because satellite cells have already differentiated during the time of cell culture or because only very few satellite cells were present initially). In contrast, no positive labelling at all for the haemopoietic stem cell marker CD34 was observed, similar to the human data shown by Alessandri and colleagues [26]. In summary, the cells injected into the sphincter were, conservatively estimated, to at least ~50% of myogenic origin. The remainder, especially as no preplating or sorting was performed, could very well be of fibroblast origin. This, per se, may not be a disadvantage as a mixture of these two connective tissue cells, both known to actively produce extracellular matrix, may result in the formation of a both structurally robust architecture and a mechanically functional tissue. It seems logic that cells for clinical applications should have a high regenerative potential; however, it is not known whether single or multiple injections would be sufficient to achieve a stable functional improvement over a given time period. Thus, our results with a responding rate of about 54% and a continence rate of 12% after 1-year follow-up do not surprise. In contrast, moreover results with highest response and continence rates after short follow-up as frequently reported in current literature have to be critically analysed. Carr and colleagues, although in women, observed a clinical effect of the transplanted cells after 3–8 months which is in accordance to the 4.7 months with earliest signs of improvement seen here [10]. 

This study has various limitations. The most important challenge in this observative setting was to assess treatment success. The initially planned questionnaire was not replied by the majority of patients, thus a less complicated modality had to be used for getting minimal information about treatment success. Abdel-fattah and colleagues found out that simply asking a patient about continence after a procedure was as good as doing the pad test and even correlated better with the patient quality of life [27]. Thus, we assume justification for our approach of evaluating treatment outcome. In addition, we did not perform posttherapy re-evaluation in terms of urethrocystomannometry. However, as declared initially, this has been an “individual treatment attempt” which did not oblige the patient to any additional examination (invasive or non-invasive). Various reasons for nonresponding to cell therapy are possible such as false injection with leakage of cells from the puncture site, lack of engraftment due to adjacent scar tissue, infection with leukocyte infiltration and local acidosis, or simply the number of implanted may have been to small, the respective sphincter damage may have been too huge. 

## 5. Conclusion

Transplantation of autologous skeletal myoblasts to repair the function of the injured external urethral sphincter was easy and safe and seems to have moderate clinical benefit. The cells injected into the sphincter were to at least ~50% of myogenic origin. Clinical trials using MDCs for the treatment of urinary incontinence are warranted. 

## Figures and Tables

**Table 1:** Patient characteristics at baseline.

Age	
Mean age	70 (56–81)
Previous surgery	
Radical prostatectomy	192 (87%)
TUR-P	9 (4%)
Radical cystectomy with neobladder	21 (9%)
Previous therapy approaches	
Pelvic floor muscle training (PFMT)	178 (80%)
Transcutaneous electrical nerve stimulation	32 (14%)
Behavioural therapies	66 (30%)
Pharmacologic treatment (duloxetine)	182 (82%)
Duration of previous incontinence	
Overall months	49 (12–192)
12–24 months	69 (31%)
24–50 months	117 (53%)
50–100 months	32 (14%)
100–192 months	4 (2%)

## Abbreviations

MDC: Muscle-derived cells
MPC: Muscle progenitor cells
SUI: Stress urinary incontinence
SOP: Standard operating procedure
GMP: Good medical practice
GCP: Good clinical practice
AMG: Arzneimittelgesetz (German Medicinal Law)
DFG: Deutsche Forschungsgemeinschaft
NRW: Nordrhein Westfalen.
